# Behçet's Disease and Intracardiac Thrombosis: A Report of Three Cases

**DOI:** 10.1155/2013/637015

**Published:** 2013-07-02

**Authors:** Nurşen Düzgün, Orhan Küçükşahin, Kayhan Çetin Atasoy, Canan Togay Işıkay, Demet Menekşe Gerede, Ayşe Erden, Seda Kaynak Şahap, Muhammed Arif İbiş, Aşkın Ateş

**Affiliations:** ^1^Department of Internal Medicine-Rheumatology, Ankara University, Faculty of Medicine, Akademik Yerleşke, Sıhhıye, 06100 Ankara, Turkey; ^2^Department of Radiology, Ankara University, Faculty of Medicine, Akademik Yerleşke, Sıhhıye, 06100 Ankara, Turkey; ^3^Department of Neurology, Ankara University, Faculty of Medicine, Akademik Yerleşke, Sıhhıye, 06100 Ankara, Turkey; ^4^Department of Cardiology, Ankara University, Faculty of Medicine, Akademik Yerleşke, Sıhhıye, 06100 Ankara, Turkey; ^5^Department of Internal Medicine, Ankara University, Faculty of Medicine, Akademik Yerleşke, Sıhhıye, 06100 Ankara, Turkey

## Abstract

We present three patients with Behçet's disease associated with intracardiac thrombus and pulmonary vascular involvement. One of these patients had also Budd-Chiari syndrome. All patients were treated with corticosteroid plus monthly intravenous cyclophosphamide as first line treatment and with no recurrences. Immunosuppressive therapy was successful in the treatment of intracardiac thrombus and also in the regression of pulmonary vascular thromboses in these patients. Intracardiac thrombus in Behçet's disease is rarely seen. Behçet's disease should be remembered in the differential diagnosis of the patients with intracardiac mass, especially in patients from the Mediterranean and Middle East populations.

## 1. Introduction

Behçet's disease (BD) is a multisystemic, chronic, inflammatory disorder of unknown etiology. In 1937, Dr. Behçet first described the disease as a distinct clinical picture with a triple complex of recurrent oral aphthosis, genital ulcer, and iridocyclitis with hypopyon [1]. BD may also involve other locations such as skin, joint, central nervous system, gastrointestinal tract, lung, and cardiovascular system. The diagnosis is primarily based on clinical criteria because there is no specific diagnostic laboratory test. The histopathologic findings are also nonspecific. BD is frequent among the Mediterranean, Middle East, and Far Eastern populations.

 Behçet's disease is recognized as a systemic vasculitis involving both arteries and veins of any size. Vascular involvement has occurred in one-third of patients. Most vascular events consist of recurrent superficial or deep vein thrombosis [2–4]. Arterial thrombosis is less frequent [3].

The cardiac findings were found in 1%–6% of BD cases in previous clinical series [5] and in 16.5% of cases in the Japanese autopsy registry [6]. Cardiac manifestations of BD include endocarditis, myocarditis, pericarditis, endomyocardial fibrosis, coronary arteritis with or without myocardial infarction, aneurysms of the coronary arteries, valve dysfunction, conduction system disturbances, and intracardiac thrombosis (ICT) [5, 7–9]. ICT is a rare manifestation of the disease [10–12]. 

 Here we present the characteristic clinical findings in three cases with ICT related to BD and review the approach to treatment of ICT in BD patients.

## 2. Patient 1 

A 22-year-old Turkish male presented to the emergency ward with complaints of fever, hypoesthesia on the left arm, and slurred speech that had lasted for a week. Medical history revealed previous attacks of intermittent fever together with recurrent oral and genital ulcers, abdominal pain, and lack of appetite. The complaints occurred once a month for the past three years and lasted for a few days. On admission, his body temperature was 39.5°C. Physical examination revealed oral and genital ulcers and a bilateral decrease in pulmonary breath sounds. Neurological examination revealed right facial paralysis and dysarthria. There were no motor and sensory deficits. 

Laboratory results were as follows: hemoglobin 9.2 gr/dL, white blood cell (WBC) 11 × 10^9^/L, platelets 200 × 10^9^/L, erythrocyte sedimentation rate (ESR) 120 mm/h, C-reactive protein (CRP) 180 mg/L, and serum albumin 2.5 g/dL. Renal and hepatic functions were normal.

**Figure 1:**
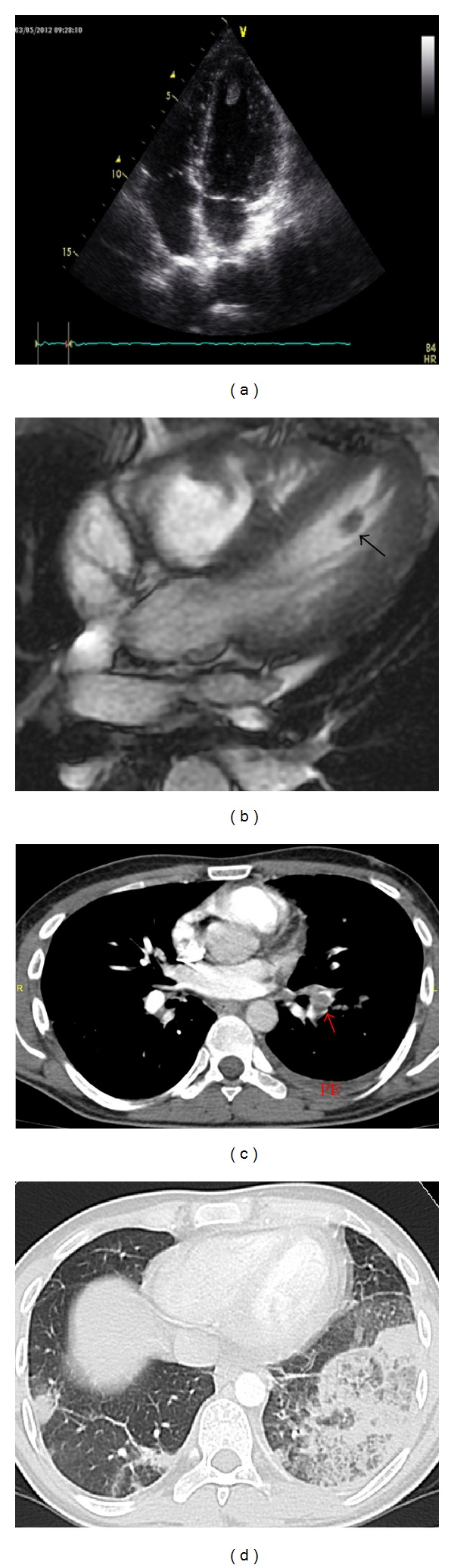
(a) Transthoracic echocardiographic apical four-chamber view shows a thrombus in the apex of the left ventricle. (b) Four-chamber-view MR image obtained with fast imaging steady-state free precession sequence shows pedunculated thrombus (arrow) attached to left ventricular apex with a thin stalk (cine images showed mobility of the thrombus throughout the cardiac cycle). (c) Pulmonary CT angiography shows acute embolus as a filling defect in the left lower lobe artery (arrow). Note the associated left pleural effusion (PE). (d) Lung window setting shows bilateral lower lobe infarcts, more prominent in the left lung.

Diffusion-weighted images of the brain demonstrated multiple acute infarcts in the right frontal, parietal, temporal, and occipital regions and right caudate nucleus. Ultrasonographic imaging of the carotid and vertebral arteries, and angiography of the brain were unremarkable. The patient was referred to the stroke unit of the department of neurology. Transthoracic echocardiography revealed a mobile hyperechogenic mass with 12 × 11 mm in size on the apex of the left ventricle (Figure 1(a)). Low molecular weighted heparin and low dose acetylsalicylic acid were started based on the diagnosis of multiple cerebral infarcts due to possible cardioembolism. 

The patient was empirically started on wide spectrum antibiotics including ceftriaxone and clarithromycin. Fever persisted under antibiotherapy. Microbiological analyses were negative. The patient was referred to the rheumatology clinic with suspicion of systemic vasculitis. Autoimmune serology (antinuclear antibodies, ANCA, and antiphospholipid antibodies) was all negative. 

Cardiac magnetic resonance (MR) showed a thrombus in the left ventricle (Figure 1(b)). Pulmonary CT angiography showed multiple acute emboli and infarctions affecting both lungs and bilateral minimal pleural effusion (Figures 1(c) and 1(d)). 

Hereditary thrombotic risk factors including prothrombin gene 20210G-A and factor V Leiden gene mutations were negative. Methylene tetrahydrofolate reductase (MTHFR) mutation was found homozygote positive.

The patient's history of recurrent oral and genital ulcers, the presence of the scrotal ulceration scars, cerebral and pulmonary vascular lesions, and cardiac thrombus supported the diagnosis of BD. There was no evidence of ocular involvement, and the Pathergy test was negative.

The patient was given intravenous high dose methylprednisolone (1 gr/d for three days), followed by oral prednisone (1 mg/kg/d), monthly intravenous cyclophosphamide (500 mg/m^2^/for one day), and colchicine (1.5 mg/d). The fever responded well to the corticosteroid and immunosuppressive therapy. 

After three months, the intracardiac thrombus was no longer detectable on echocardiography. The neurological examination was unremarkable. Nine months later, the patient was asymptomatic and complete resolution of ICT persisted. 

## 3. Patient 2 

 A 24-year-old Turkish male with a previous diagnosis of BD for 10 years was admitted to the rheumatology clinic following a 1-month history of fever and dyspnea on exertion.

On physical examination, body temperature was 38°C. Pulmonary sounds were bilaterally decreased. Laboratory results were as follows: hemoglobin 11.1 gr/dL, WBC 10.8 × 10^9^/L, platelets 280 × 10^9^/L, ESR 103 mm/h, and CRP 148 mg/L. Renal and hepatic functions were normal. Microbiologic studies did not identify any causative organisms.

**Figure 2:**
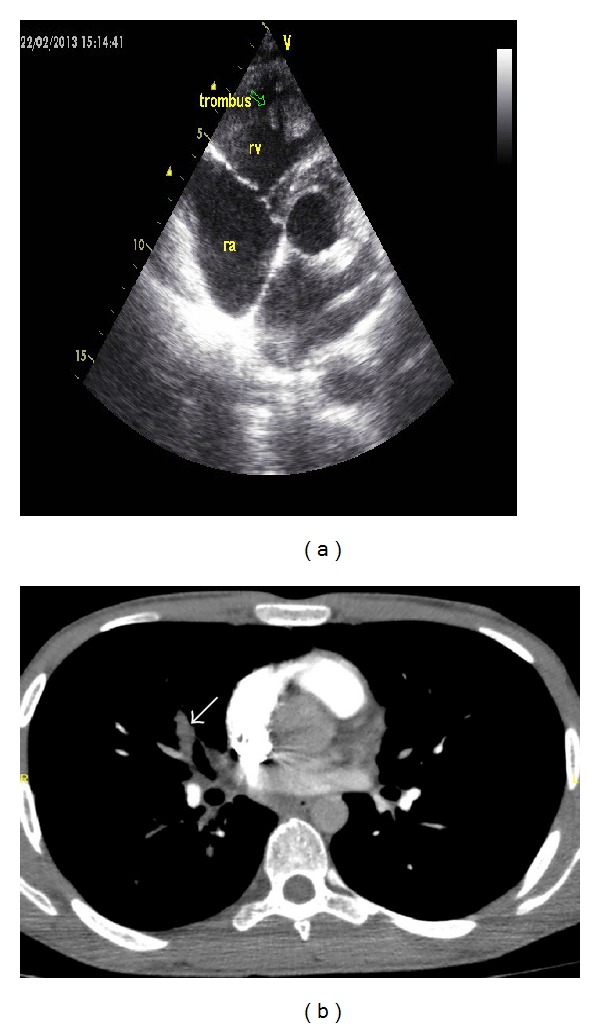
(a) Transthoracic echocardiography in the parasternal short-axis view shows a thrombus (arrow) in the right ventricle attached to the interventricular septum. (b) Contrast-enhanced pulmonary CT angiography shows acute embolus filling and mildly enlarging the right middle lobe artery (arrow).

Echocardiography revealed a mobile mass (13 × 12 mm in size) in the right ventricle attached to the interventricular septum (Figure 2(a)).

Pulmonary CT angiography showed filling defects due to acute embolism in the middle lobe arteries, apical and posterior basal segment arteries of the right lower lobe, as well as small subpleural consolidations in the right middle and lower lobes representing infarcts (Figure 2(b)). 

 HLA-B51 and Pathergy tests were negative. Hereditary thrombotic risk factors including prothrombin gene 20210G-A, factor V Leiden and MTHFR gene mutations were negative. Ocular examination showed sequel uveitis in the left eye. The patient was given intravenous high dose methylprednisolone (1 gr/d for three days), followed by oral prednisone (1 mg/kg/d), and monthly intravenous cyclophosphamide (500 mg/m^2^/for one day) under a diagnosis of BD. Dyspnea and fever regressed with the corticosteroid and immunosuppressive therapy. After nine months, the intracardiac thrombus showed complete resolution on echocardiography.

The patient was continued to be treated with monthly cyclophosphamide, prednisone, and colchicine.

## 4. Patient 3 

A 25-year-old Turkish man who had been followed for one year with a diagnosis of Budd-Chiari syndrome was admitted to the rheumatology clinic with fever up to 40°C and abdominal pain. On admission, his body temperature was 39°C. Physical examination revealed prominent superficial collateral veins in the chest wall and scars of old ulcers in the scrotum and also papulopustular lesions in the extremities and neck.

Laboratory tests on admission revealed WBC of 6.6 × 10^9^/L, hemoglobin of  13.1 g/dL, ESR of 36 mm/h, and CRP concentration of 43.5 mg/dL. Urinalysis, kidney, and liver functions were normal. Microbiologic studies were found negative. Autoimmune serologic tests were negative. He was started on tazocin (piperacillin and tazobactam) and clarithromycin as empiric antibiotherapy, but fever persisted under this therapy.

**Figure 3:**
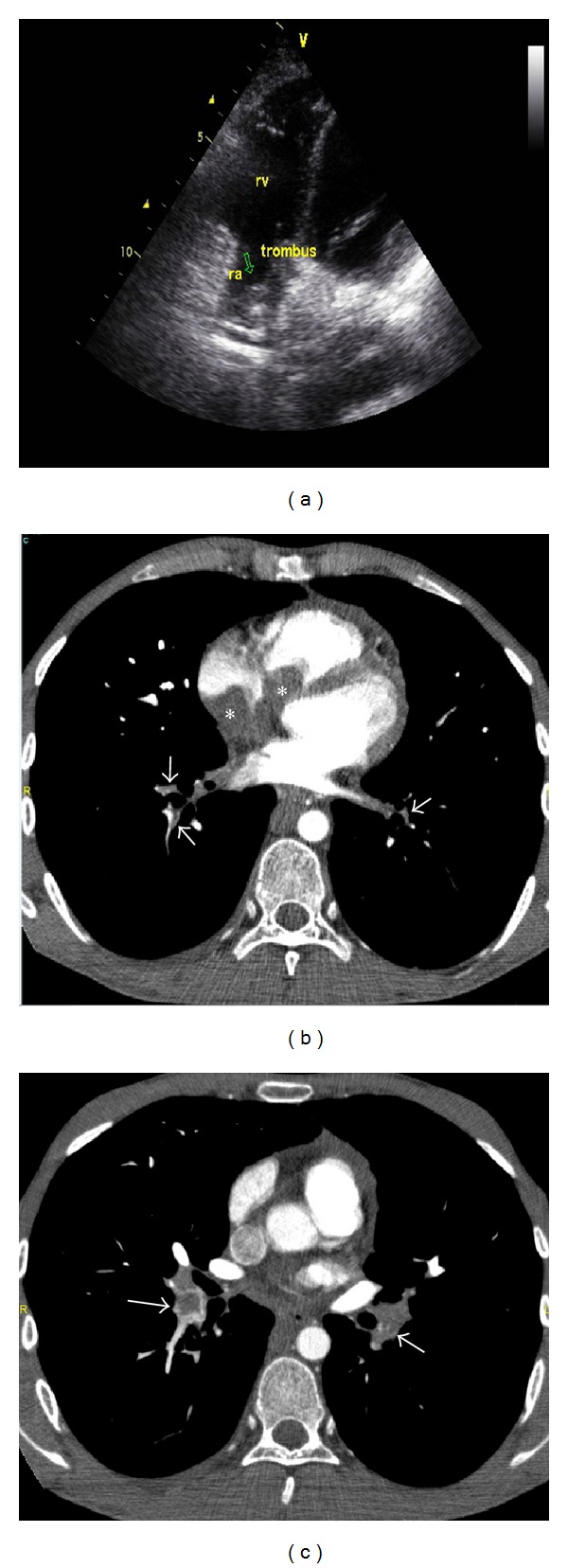
(a) Transthoracic echocardiography showed a mobile mass filling the half of the right atrium (4.5 × 3 cm in size) (arrow) extending the right ventricule (1.5 × 1.6 cm in size) and into the vena cava. (b) Contrast-enhanced CT angiographic image shows a large filling defect in the right atrium extending into the right ventricle (asterisks). Segmental arteries in both lower lobes are of small calibre due to chronic thromboembolism (arrows). Pulmonary hypertension resulted in straightening of the interventricular septum. (c) Both lower lobe arteries contain central filling defects (arrows) representing superimposed acute pulmonary embolism.

Transthoracic and transesophageal echocardiography showed a mobile mass (4.5 × 3 cm in size) filling half of the right atrium and extending into the right ventricle and inferior vena cava (Figure 3(a)). Pulmonary arterial pressure by echocardiography was 30 mmHg. 

Contrast-enhanced CT angiography confirmed the thrombus in the right atrium that extended into the right ventricle and displayed findings of both chronic and superimposed acute thromboembolism (Figures 3(b) and 3(c)). The right ventricle showed signs of pulmonary hypertension including chamber dilatation, myocardial thickening, and straightening of the interventricular septum. 

Medical history revealed complaints of recurrent oral and genital ulcers and papulopustular lesions. HLA-B51 was positive. Pathergy test was negative. The patient was diagnosed with BD based on these findings. Hereditary thrombotic risk factors including prothrombin gene 20210G-A, factor V Leiden and MTHFR gene mutations were negative. 

The patient was given methylprednisolone (1 mg/kg/d) and monthly cyclophosphamide (500 mg/m^2^ for a day). Oral anticoagulant was stopped, and low molecular weighted heparin was started. Clinical findings regressed and laboratory abnormalities returned to normal. Six months later, the ICT was decreased substantially in size. The patient was continued to be treated with monthly cyclophosphamide, prednisone, and colchicine, without recurrence. 

## 5. Discussion

Intracardiac thrombosis is an uncommon complication of BD. Most of the patients are young males and are from the Mediterranean and the Middle East. Though ICT is usually located in the right side of the heart, mainly the right ventricle, thromboses involving both ventricles or limited only to the left ventricle have been also described [10–14]. In two of our patients, ICT was located in the right side of the heart, and one had thrombus in his left ventricle. All our patients had fever and dyspnea, which were the most common presenting symptoms in previously reported cases [5, 10–15]. Sometimes ICT may be the first manifestation of the disease [10, 15].

ICT often coexists with pulmonary involvement such as pulmonary artery aneurysms [16–21] and/or pulmonary artery thrombosis/pulmonary thromboembolism [10–15, 21–24], as was the case in our patients. In a review, pulmonary thromboembolism was reported in 67% of patients with ICT [11].

ICT has been also frequently associated with superficial thrombophlebitis and deep venous thrombosis [15, 23, 24] or thrombosis of vena cava [11], as in patient 3 with Budd-Chiari syndrome, which is an unusual disorder characterised by hepatic venous outflow obstruction either within the liver or in the inferior vena cava. In a study, of 493 patients with BD, 14 patients had Budd-Chiari Syndrome (2.8%) [25]. Association of ICT with Budd-Chiari syndrome seems to be rare. There have been only two cases including our case in the literature [26].

It has been speculated that pulmonary embolism or pulmonary infarction might have originated from deep vein thrombosis or right ventricular thrombi [20, 26–28]. Histopathological studies reveal thrombi and inflammatory infiltrates in vessel walls, which addresses a vasculitic process. Pulmonary vascular inflammation may result in thrombosis, infarction, hemorrhage, or aneurysm. There is no risk of embolic events in BD associated with deep vein thrombosis which is a feature of inflammation and also thrombus strictly adheres to inflamed vessel wall [29–31]. Pulmonary vascular manifestations are a result of in situ pulmonary pathology rather than embolisation from systemic veins [10].

The pathogenetic mechanism of thrombus formation in BD is still unclear. It is attributed to vascular inflammation and endothelial ischemia. High levels of endothelial products such as Von Willebrand factor antigen support endothelial destruction due to vasculitis [32]. Genetic prothrombotic factors such as Factor V Leiden and Prothrombin 20210 G-A gene mutations in BD were found to be markedly increased for the risk of thrombosis [33, 34]. A meta-analysis showed that hyperhomocysteinemia may be associated with thrombosis in BD [35]. Endomyocardial fibrosis may be one of the causes of ICT formation [10].

ICT can be misdiagnosed as an intracardiac tumor such as myxoma or vegetation related to infective endocarditis. Imaging tests such as echocardiography, CT, or MR show the filling defect(s) in the chamber(s). CT and MR also provide information about pulmonary vascular and/or parenchymal lesions. In most cases, ICT is discovered by echocardiography in the workup of fever etiology such as bacterial endocarditis.

Compared with transthoracic echocardiography, cardiac MR imaging is found to be more sensitive for the detection of left ventricular thrombi. The results of the same study also showed that ECG-triggered contrast-enhanced MR imaging is an accurate noninvasive alternative technique for the characterization of intracardiac thrombi, which is important because clots have a higher risk of embolism than that of organized thrombi [36]. 

There is no consensus for the treatment regimes of ICT associated with BD. There is no randomised controlled study assessing the results of different therapeutic protocols. 

ICT in BD has been treated medically and occasionally with surgery and/or thrombolytic therapy. Medical treatment includes colchicine, corticosteroids, immunosuppressive agents, anticoagulants and/or low dose aspirin. It has been concluded that medical treatment should be the first choice of therapy [11, 26].

Immunosuppression is the main therapy for the treatment of vasculitis. Most cases of BD complicating ICT showed complete resolution or decreased in size with systemic corticosteroid and intravenous cyclophosphamide along with anticoagulants [5, 13, 14, 20, 26, 37, 38]. Ahn et al. showed that there were no significant differences in the recurrence rate of thrombosis between immunosuppressive therapy and those combined with anticoagulants therapy [39]. 

Immunosuppressive therapy without anticoagulation was also associated with complete resolution of the thrombus with no recurrences [40], as in our patients 1 and 2. Anticoagulation therapy can cause life-threatening massive bleeding in the presence of pulmonary aneurysm(s) in patients with ICT. Pulmonary aneurysms and ICT responded well to immunosuppressive therapy without anticoagulation [18], or the anticoagulants may be given after the resolution of hemoptysis with aggressive immunosuppressive treatment [41]. There is no clear evidence to support the benefit of anticoagulation in the treatment of thrombotic lesions in BD. Systemic corticosteroids plus immunosuppressives are highly recommended before anticoagulation in cases of BD [11, 14, 42].

Diagnostic and therapeutic cardiac surgical resection has been performed [11, 19, 28, 42–46]. Surgical treatment may be considered in cases unresponsive to optimal medical treatment, ineffective thrombolysis therapy and impaired hemodynamics or if ICT becomes massive and shows recurrence. Surgery alone does not lead to complete resolution [10, 42, 43]. There is a risk of recurrence after surgery [5]. After surgical removal of ICT, recurrent thrombus successfully resolves with medical treatment including prednisolon and immunosuppressant [22, 44–46]. Some patients underwent surgery before immunosuppressive therapy [10]. 

Thrombolytic therapy is rarely utilized in BD patients with ICT. Full resolution of thrombosis was reported with intravenous streptokinase plus corticosteroid treatment [12, 23]. A case with multiple ICTs has needed thrombolytic treatment along with anticoagulation, cyclophosphamide, and anti-TNF agent for resolution of ICT [47]. 

ICT in patients with BD may be resolved with medical treatment including immunosuppressive agents such as cyclophosphamide along with high dose glucocorticoid. Our results show that this combination therapy is the preferred choice of treatment.
